# Supplementary material for: The Chlamydia trachomatis Inc Tri1 interacts with TRAF7 to displace native TRAF7 interacting partners
Source: Microbiol Spectr. 2024 May 30;12(7):e00453-24. doi: 10.1128/spectrum.00453-24 (PMC11218536; doi:10.1128/spectrum.00453-24)
Supplement: Supplemental figures and table — Fig. S1-S5; Table S1. [file spectrum.00453-24-s0001.pdf]

Supplementary Materials for

The *Chlamydia trachomatis* Inc Tri1 interacts with TRAF7 to displace native TRAF7 interacting partners

This PDF file includes:

Supplementary Table 1 and supplementary Fig. 1 to Fig. 5.

Table S1. Primers used in this study.

Primer	Primer Sequence (5'--> 3')	Primer Use
CH_P006	GGG GCG GCC GCT CGA GTC ATG AGC TCA GGC AAG AGT GCC CGC TA	Generate FLAG-TRAF7 (pcDNA 4/TO 3X FLAG vector)
CH_P002	CAGCGGGTTTAAACGGGCCCTTAGCAAGTCCAAACCTTCACAGTG	Generate FLAG-TRAF7 (pcDNA 4/TO 3X FLAG vector)
CH_P006	GGG GCG GCC GCT CGA GTC ATG AGC TCA GGC AAG AGT GCC CGC TA	Generate FLAG-delta WD40 (pcDNA 4/TO 3X FLAG vector)
CH_P013	CAGCGGGTTTAAACGGGCCCGTAGGAGCCTAGGATGCCC	Generate FLAG-delta WD40 (pcDNA 4/TO 3X FLAG vector)
CH_P014	GGGGCGGCCGCTCGAGTCGACCCTCAGCAGATCTTCAAGTG	Generate FLAG-WD40 (pcDNA 4/TO 3X FLAG vector)
CH_P002	CAGCGGGTTTAAACGGGCCCTTAGCAAGTCCAAACCTTCACAGTG	Generate FLAG-WD40 (pcDNA 4/TO 3X FLAG vector)
CH_P015	GGGGCGGCCGCTCGAGTCGAGATGCACGTGGCTCTGG	Generate FLAG-cc + WD40 (pcDNA 4/TO 3X FLAG vector)
CH_P002	CAGCGGGTTTAAACGGGCCCTTAGCAAGTCCAAACCTTCACAGTG	Generate FLAG-cc + WD40 (pcDNA 4/TO 3X FLAG vector)
CH_P003	TCTCGAGCTCAAGCTTCGATGAGCTCAGGCAAGAGTG	Generate mCh-TRAF7 (pmCherry-C1 vector)
CH_P004	GATCCCGGGCCCGCGGTACCTTAGCAAGTCCAAACCTTCACAGTG	Generate mCh-TRAF7 (pmCherry-C1 vector)
CH_P003	TCTCGAGCTCAAGCTTCGATGAGCTCAGGCAAGAGTG	Generate mChTRAF7-delta WD40 (pmCherry-C1 vector)
CH_P016	GATCCCGGGCCCGCGGTACCGTAGGAGCCTAGGATGCCC	Generate mChTRAF7-delta WD40 (pmCherry-C1 vector)
CH_P017	TCTCGAGCTCAAGCTTCGGACCCTCAGCAGATCTTCAAGTG	Generate mCh-TRAF7 WD40 (pmCherry-C1 vector)
CH_P004	GATCCCGGGCCCGCGGTACCTTAGCAAGTCCAAACCTTCACAGTG	Generate mCh-TRAF7 WD40 (pmCherry-C1 vector)
CH_P007	CAGTGTGGTGGAATTCATGAGTTTTGTTGGAGATAGTGTAC	Generate Tri1-Strep (pcDNA 4/TO 2X Strep)
CH_P010	CTCCCTCGAGCGGCCGCCAATCATTGGGAAAAATTGAGTGTAGA	Generate Tri1-Strep (pcDNA 4/TO 2X Strep)
CH_P007	CAGTGTGGTGGAATTCATGAGTTTTGTTGGAGATAGTGTAC	Generate Tri1 1-117-Strep (pcDNA 4/TO 2X Strep)
CH_P011	CTCCCTCGAGCGGCCGCCAAAGTCTCTGCTTATCCAAAAAG	Generate Tri1 1-117-Strep (pcDNA 4/TO 2X Strep)
CH_P007	CAGTGTGGTGGAATTCATGAGTTTTGTTGGAGATAGTGTAC	Generate Tri1 1-128-Strep (pcDNA 4/TO 2X Strep)
CH_P012	CTCCCTCGAGCGGCCGCCAAGCTAACGCGATTTCTTCTTTAG	Generate Tri1 1-128-Strep (pcDNA 4/TO 2X Strep)
CH_P009	CAGTGTGGTGGAATTCATGCTTACTAGAATTTCTGGCTATG	Generate Tri1 -84-147-Strep (pcDNA 4/TO 2X Strep)
CH_P010	CTCCCTCGAGCGGCCGCCAATCATTGGGAAAAATTGAGTGTAGA	Generate Tri1 -84-147-Strep (pcDNA 4/TO 2X Strep)
CH_P019	GGATCAAATTGGACCTGCTTGGAAGCAAGTTCACGTCC	MEKK3 mutagenesis primers (kinase dead to WT, pcDNA3.1 Myc vector)
CH_P020	GGACGTGAACTTGCTTCCAAGCAGGTCCAATTTGATCC	MEKK3 mutagenesis primers (kinase dead to WT, pcDNA3.1 Myc vector)

Figure S1.

```
CLUSTAL O(1.2.4) multiple sequence alignment


Tri1 (Serovar D)    MSFVGDSVPLRSYMPEAPLVDSASKARVSCCSERIAVLALGILSILFIVTGAALFIGAGW      60
Tri1 (Serovar L2)   MSFVGDSVPLRSYMPEAPLVDSASKARVSCCSERIAVLALGILSILFIVTGAALFIGAGW      60
                    ************************************************************

Tri1 (Serovar D)    TTLPMIDVVVTLVVFGSVMLGAVLTRISSYGGEPKKVSLDRFVLENERQGFLDKQRLADI      120
Tri1 (Serovar L2)   TTLPMINVVVTLVVFGSVMLGAVLTRISGYGGEPKKVSLDRFVLENERQGFLDKQRLADI      120
                    ******:*********************.*******************************

Tri1 (Serovar D)    SKEEIALAKQQIEEEKEAILHSIFPND       147
Tri1 (Serovar L2)   SKEEIALAKQQIEEEKEAILHSIFPND       147
                    ***************************
```

Fig S1.Alignment of Tri1 from L2 serovar D.
A. Clustal alignment(64) of Tri1 from C. trachomatis serovars D and L2. Key: * , identical residues. :, highly conserved residues, ., weakly conserved residues.

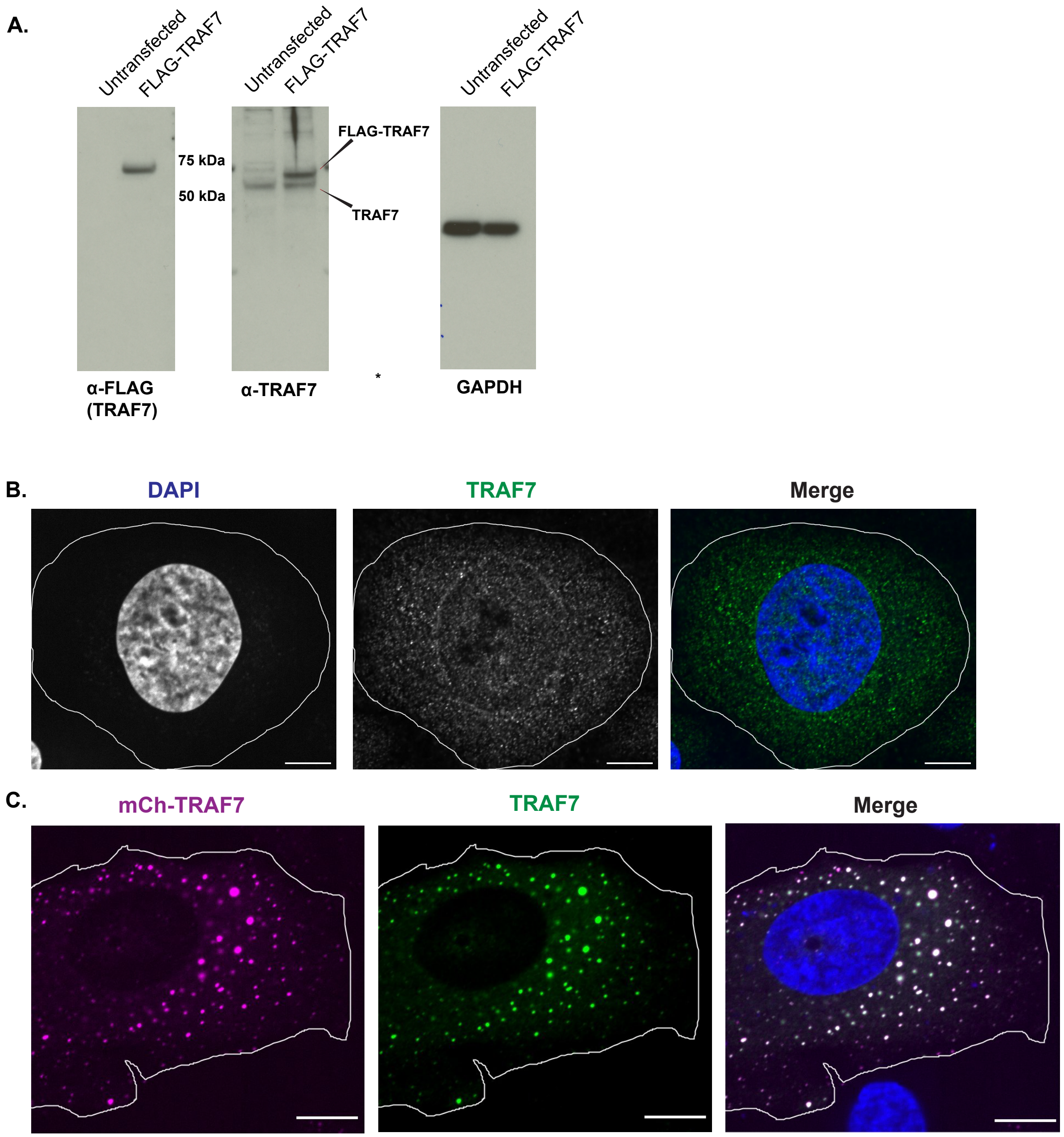

Figure S2. TRAF7 antibody detects endogenous TRAF7 and ectopically expressed TRAF7. A. Lysates prepared from a HeLa cell line that only expresses TRAF7 isoform 2 (see materials and methods) that were either untransfected or transfected with FLAG-TRAF7 were immunoblotted with the indicated antibodies. Antibodies to FLAG and TRAF7 detect similar sized proteins in the transfected lysates, which correspond with the predicted size of FLAG-TRAF7 (~78 kDa). The faster migrating band seen in the immunoblots probed with anti-TRAF7 represents endogenous TRAF7 isoform 2 (~67 kDa). GAPDH serves as a loading control. B. Hela cells that were transfected with mCh-TRAF7 for 24 hrs were fixed and stained with anti-TRAF7 and DAPI and imaged by fluorescence confocal microscopy. B. Endogenous TRAF7 localizes to both the nucleus and cytosol. Cell membrane is outlined. Shown are Single z-slices. Scale bar = 10 µm.

Uncropped blots corresponding to Fig. 1

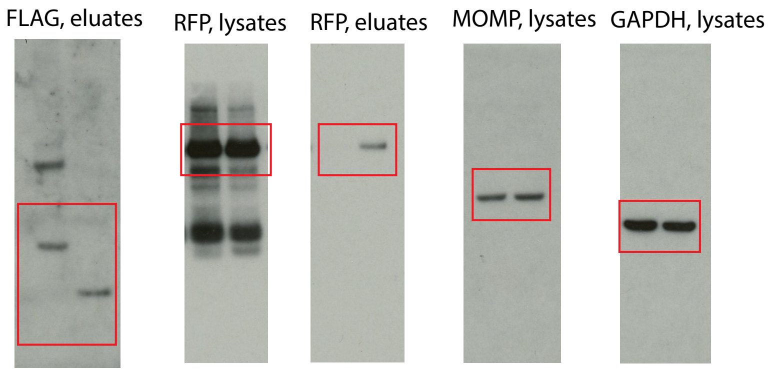

Fig S3. Uncropped immunoblots corresponding to Figure 1.

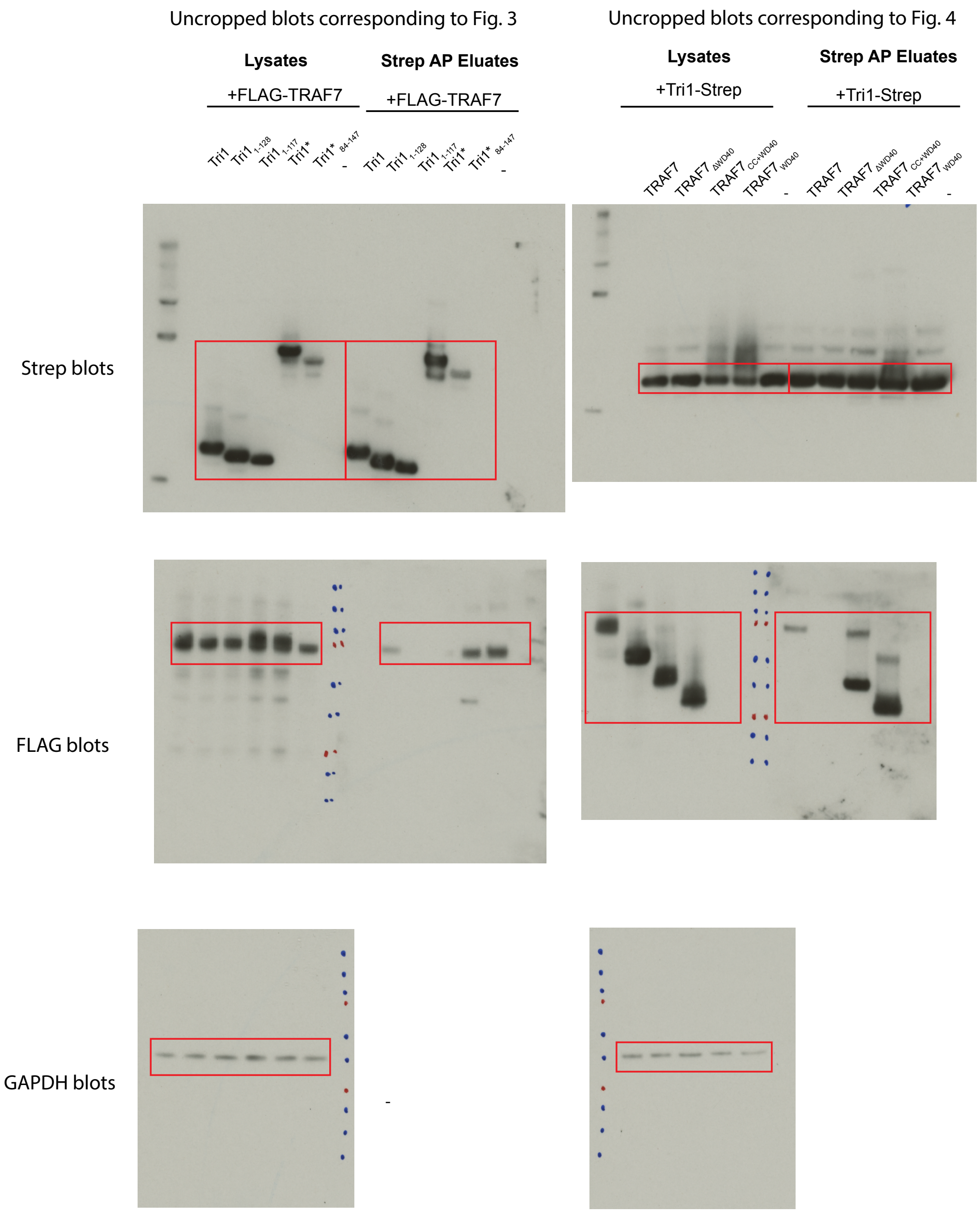

Fig S4. Uncropped immunoblots corresponding to Figures 3 and 4.

Uncropped blots corresponding to Fig. 5C

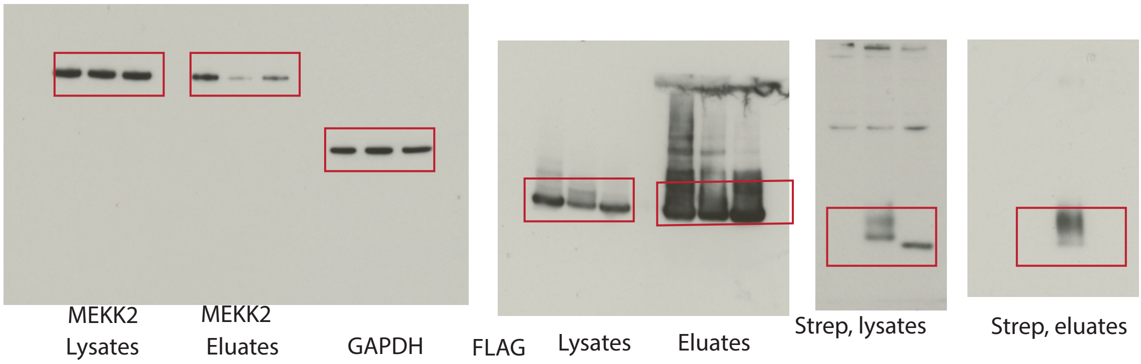

Uncropped blots corresponding to Fig. 5D

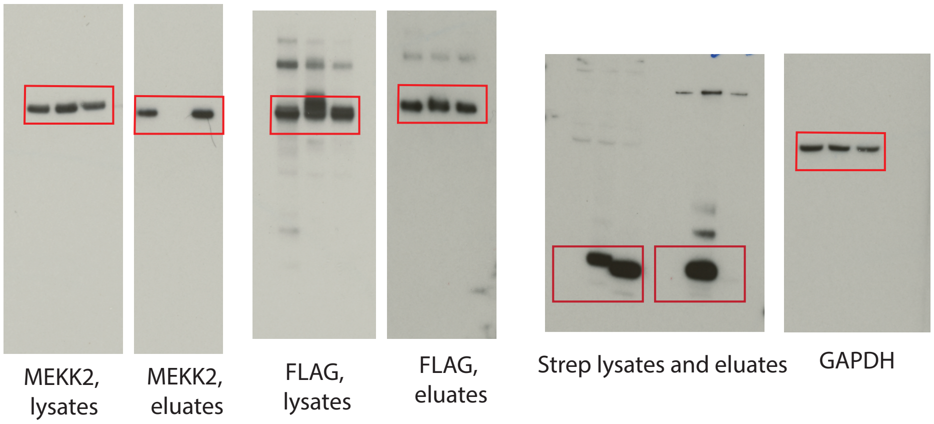

Uncropped blots corresponding to Fig. 5E

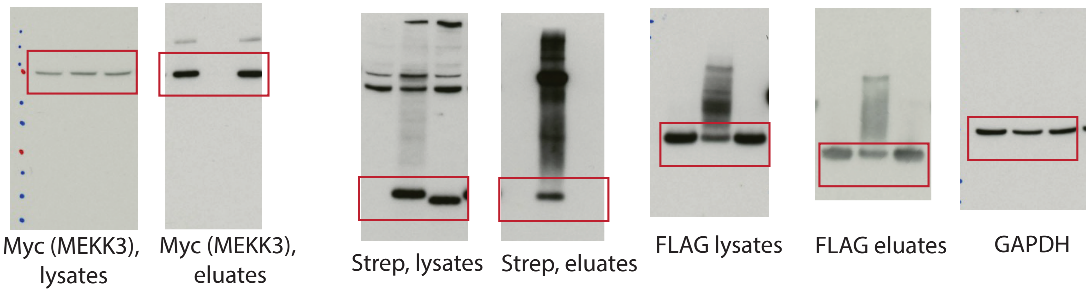

Fig S5. Uncropped immunoblots corresponding to Figure 5.
